# Supplementary material for: COVIDisgust: Language processing through the lens of partisanship
Source: PLoS One. 2022 Jul 21;17(7):e0271206. doi: 10.1371/journal.pone.0271206 (PMC9302854; doi:10.1371/journal.pone.0271206)
Supplement: S1 File — (DOCX) [file pone.0271206.s001.docx]

**Appendix 1. The list of experimental stimuli**

# **Words**

*lush*

*pregnancy*

*boy*

*powerful*

*sarcasm*

*tribunal*

*hulk*

*treat*

*tawny*

*celebrity*

*reproach*

*octopus*

*hoot*

*kite*

*saturated*

*damn*

*humbug*

*abundance*

*flesh*

*lemon*

*elf*

*opponent*

*soot*

*rebellion*

*honest*

*interested*

*rascal*

*weird*

*mind*

*reversal*

*speck*

*gnome*

*howl*

*bogus*

*failure*

*unworthy*

*annoy*

*unlucky*

*unkind*

*divorce*

*phony*

*grumpy*

*fugitive*

*disappointment*

*discontent*

*punishment*

*irritating*

*unhappy*

*unlawful*

*stingy*

*insult*

*displeasure*

*unhealthy*

*guilt*

*criticize*

*lonely*

*inconsiderate*

*bummer*

*disappointed*

*lawsuit*

*scold*

*unsatisfied*

*unfriendly*

*ungrateful*

*wimpy*

*inconvenient*

*slavery*

*germ*

*repulsive*

*suicidal*

*antichrist*

*sickening*

*obese*

*sewage*

*vermin*

*decompose*

*vomit*

*phlegm*

*loathing*

*gory*

*gonorrhea*

*puke*

*barf*

*deadly*

*parasite*

*bloodthirsty*

*incest*

*deformed*

*poisonous*

*disease*

*discrimination*

*pervert*

*fecal*

*hatred*

*malaria*

*cancer*

*canker*

*torture*

*mutilation*

# **Non-Words**

*cyntacle*

*prawk*

*platery*

*schriking*

*rimidate*

*tassible*

*medgy*

*dap*

*clynge*

*mensible*

*blutter*

*cewl*

*fellick*

*crasp*

*metailoring*

*eluctant*

*interfate*

*firtide*

*naitching*

*thwoast*

*swumpster*

*bultching*

*mencil*

*actuority*

*pealor*

*cinegar*

*nuddler*

*urodite*

*bartless*

*holfize*

*sollock*

*jucil*

*utrensive*

*accomrogation*

*creelck*

*fagle*

*debash*

*floak*

*oxylate*

*jartly*

*lincer*

*fanely*

*thwitch*

*courve*

*spinket*

*falps*

*cornesy*

*lencis*

*sploothe*

*twiest*

*ucalos*

*glupse*

*theigh*

*nucking*

*subjerd*

*stisque*

*outnel*

*mubsicle*

*sprief*

*povenable*

*furping*

*sprynx*

*lurrage*

*gressiest*

*splowse*

*speekish*

*pracker*

*mosime*

*ulassive*

*churk*

*lewsive*

*plitable*

*schreese*

*sckumpt*

*exprate*

*spaw*

*rascan*

*throofer*

*crultch*

*rulard*

*gunple*

*sottle*

*wreigged*

*olecessity*

*cint*

*noffed*

*spreeve*

*linister*

*thrinched*

*conthote*

*kirded*

*sneafes*

*spuller*

**Appendix 2.** Headlines used in the two experimental conditions

**Headline: COVID severe**

**1 2**

**
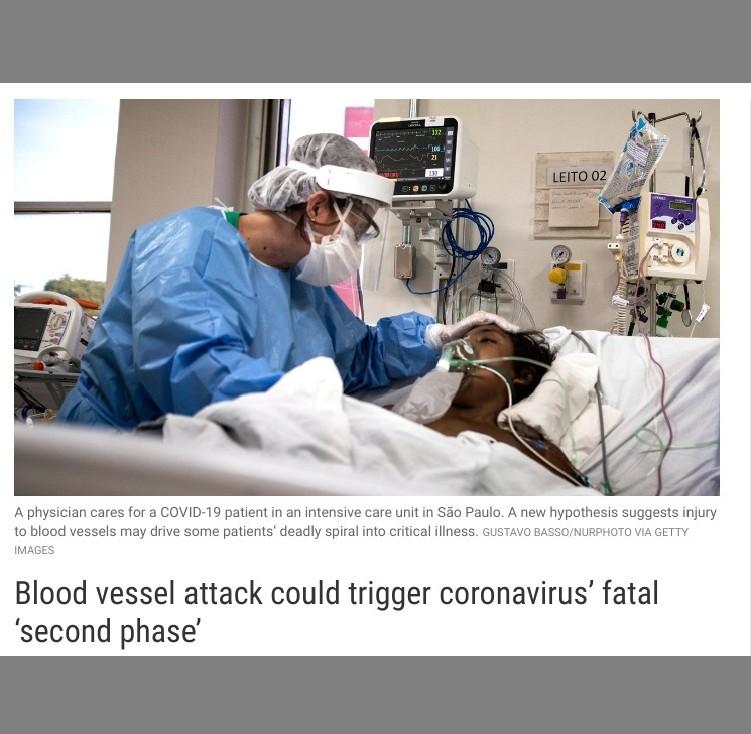

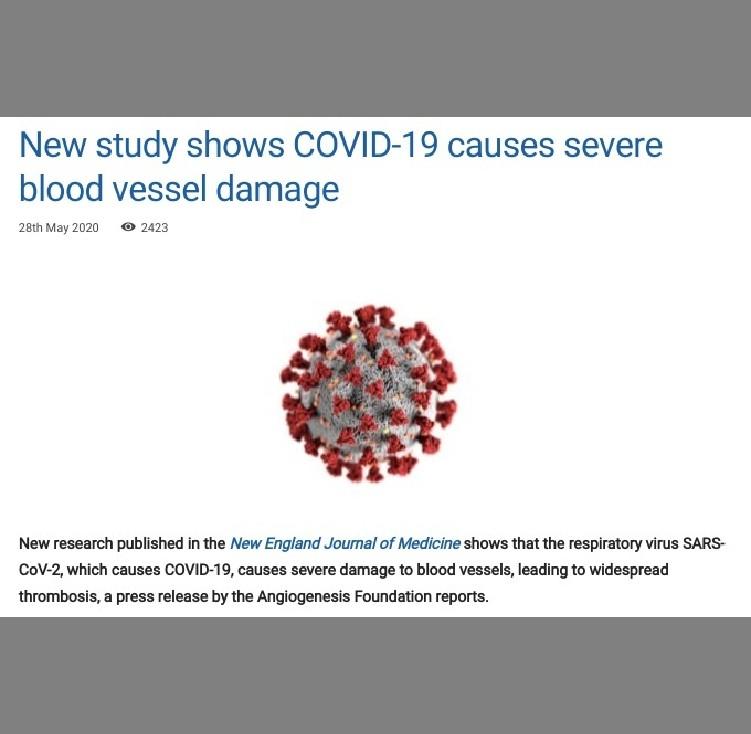
**

**3 4**

**
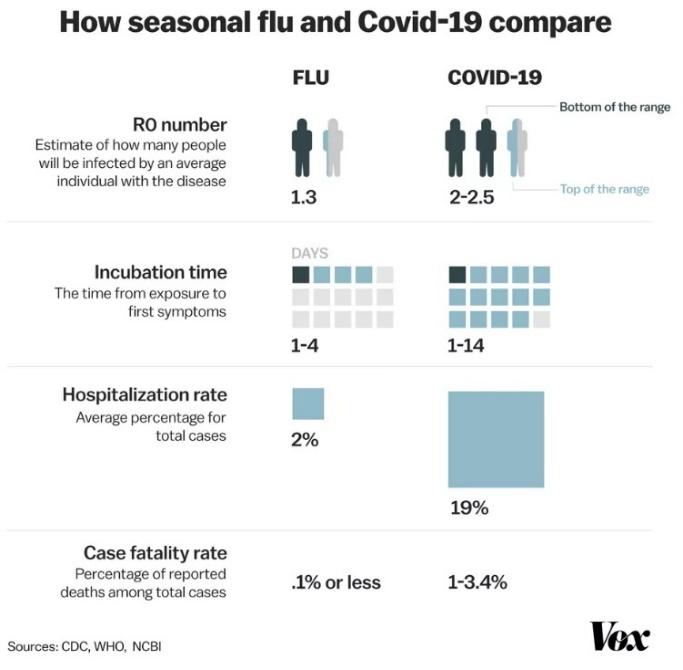

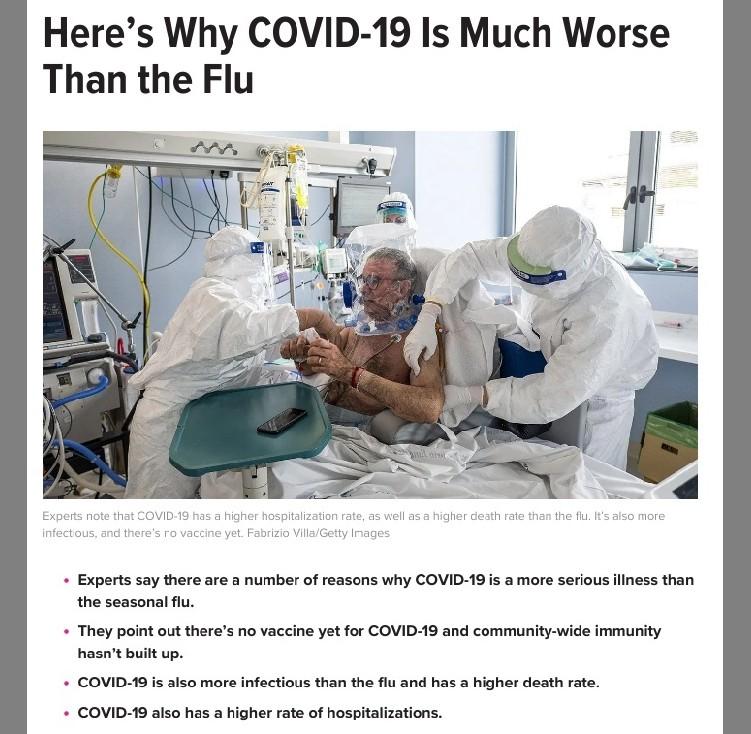
**

**5 6**

**
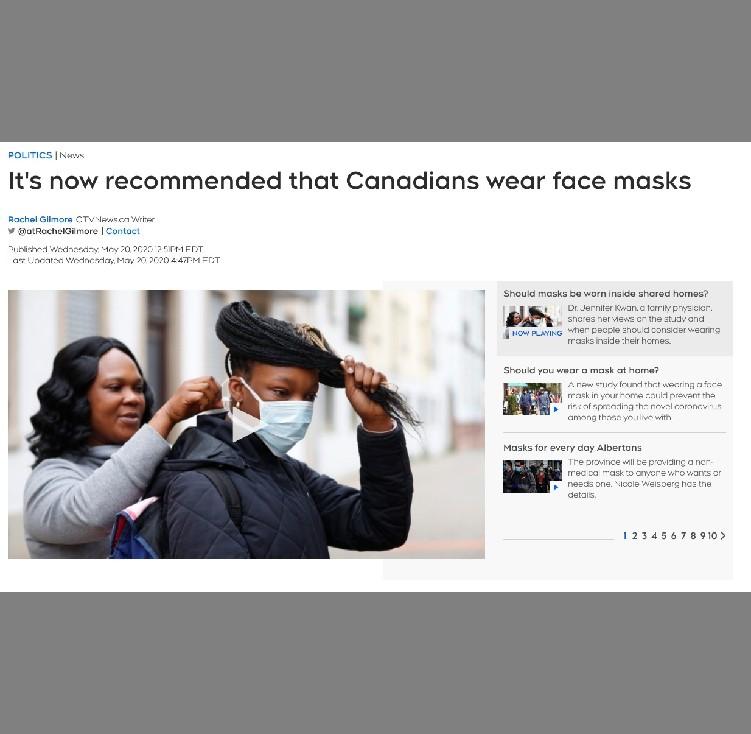

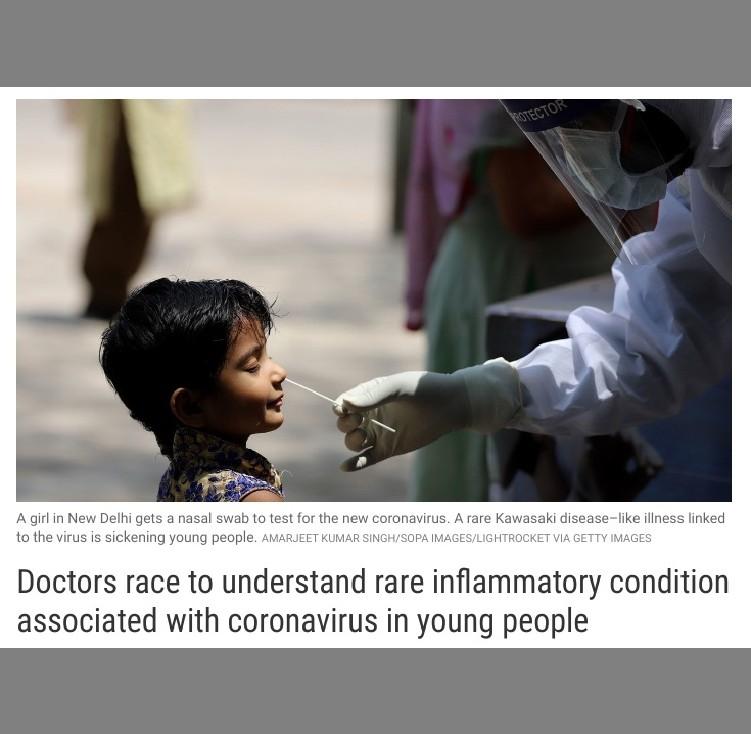
**

**7**

**
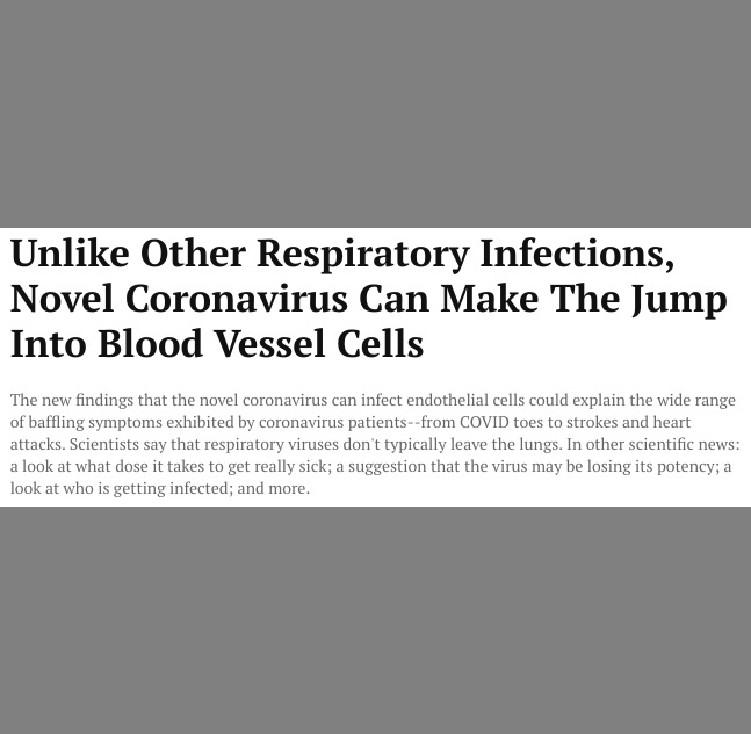
**

**Headline: COVID downplayed**

**1 2**

**
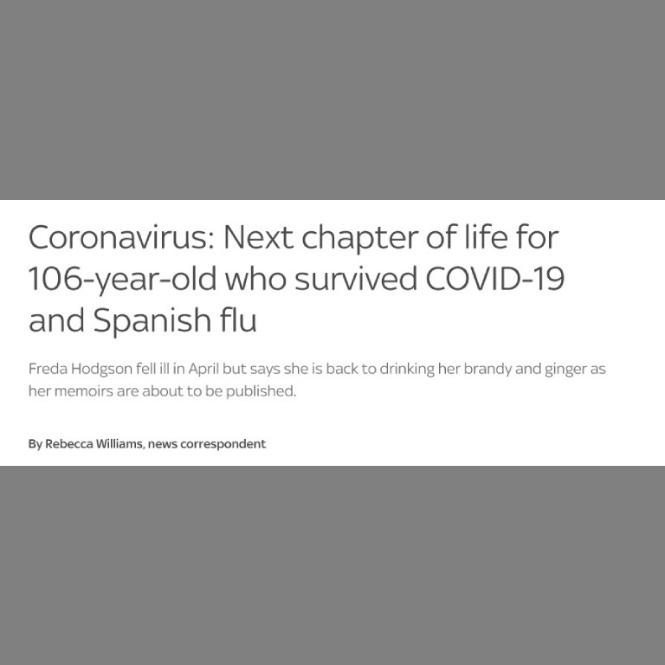

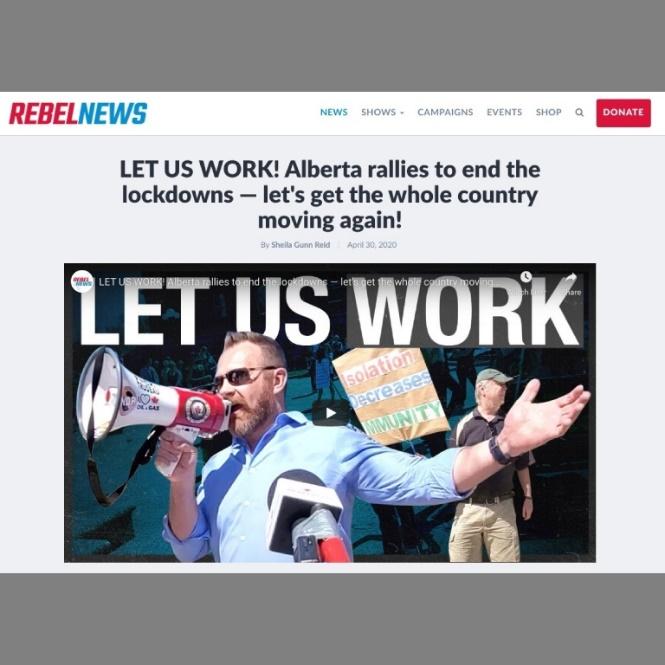
**

**3 4**

**
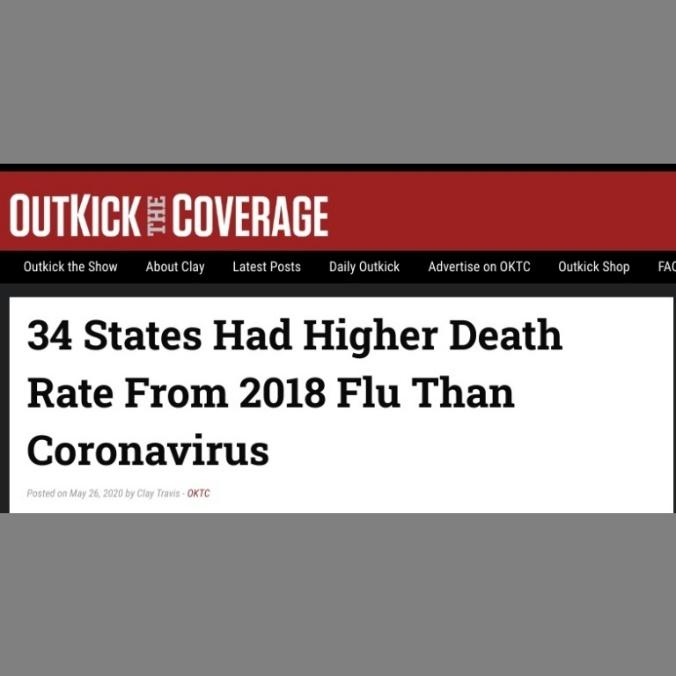

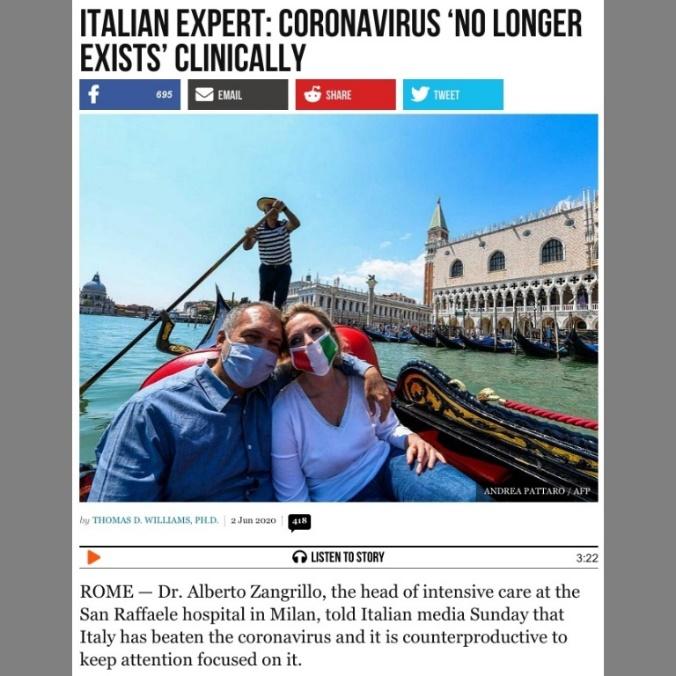
**

**5 6**

**
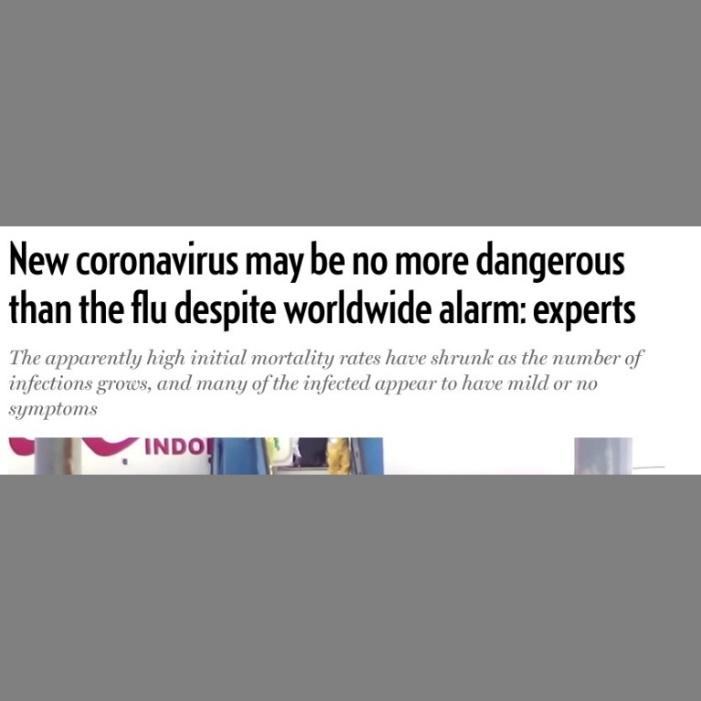

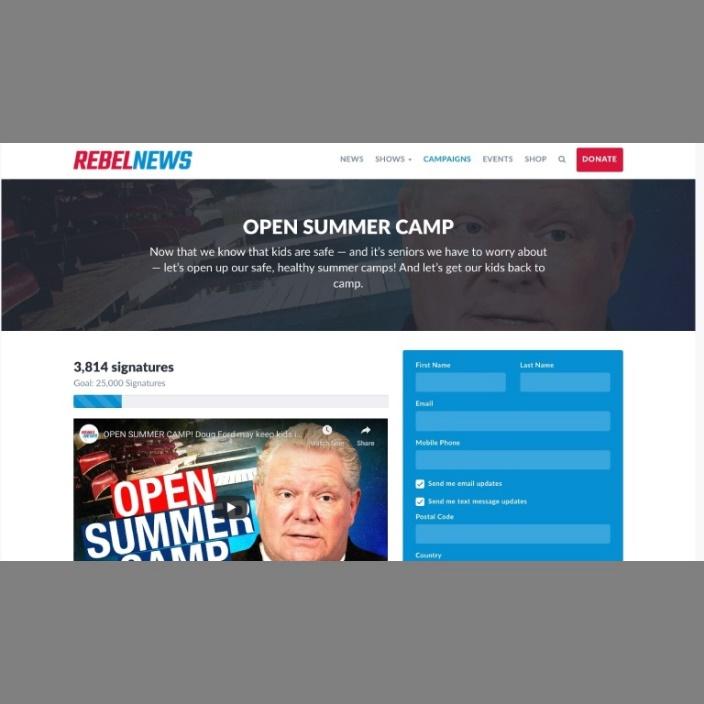
**

**7 8**

**
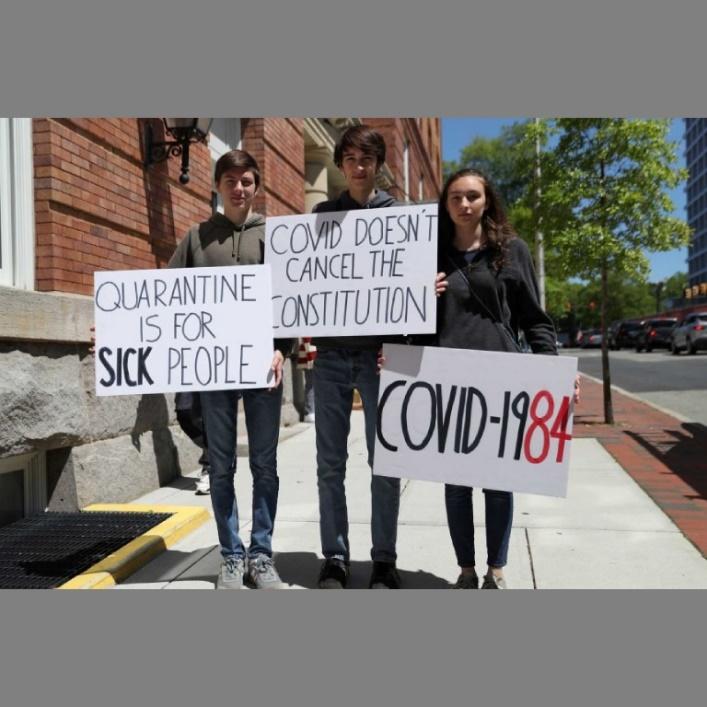

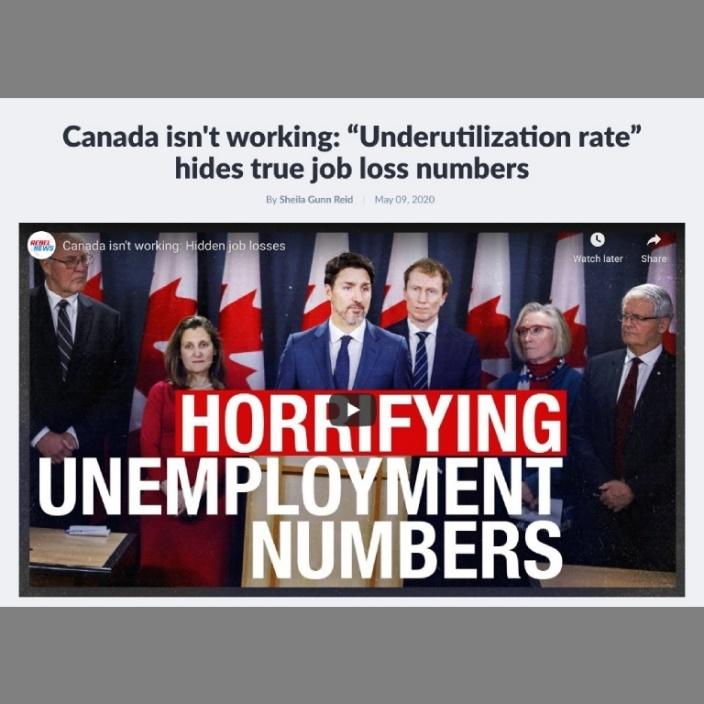
**

**Appendix 3.** LMMs for DS-R and word disgust

**Summary of the linear mixed-effects model with reciprocally transformed RTs as a dependent variable.** Model’s formula: recipr.rt ~ log.freq + length + concreteness + AoA + ns + if.match + wd.arousal + DS-R*headline*wd.disgust + (1 | participant) + (1 | word). Asterisks indicate significance (***<.001, **<.01, *<.05, .<.07).

| *Predictors* | *Estimates* | *CI* | *p* |  |  |
| --- | --- | --- | --- | --- | --- |
| (Intercept) | -844.09 | -982.07 – -706.11 | <.001 | ******* |  |
| log freq | -41.88 | -53.38 – -30.38 | <.001 | ******* |  |
| length | 6.02 | -1.37 – 13.42 | .111 |  |  |
| concreteness | 19.90 | 2.54 – 37.26 | .025 | ** |  |
| AoA | 58.85 | 40.76 – 76.95 | <.001 | ******* |  |
| ns [ns] | -193.51 | -287.77 – -99.25 | <.001 | ******* |  |
| if match [mismatch] | -24.43 | -111.12 – 62.26 | .581 |  |  |
| wd arousal | 5.46 | -26.21 – 37.13 | .735 |  |  |
| wd disgust | -10.43 | -24.92 – 4.06 | .158 |  |  |
| headline [covid severe] | -45.63 | -132.26 – 40.99 | .302 |  |  |
| DS-R | 21.61 | -48.05 – 91.28 | .543 |  |  |
| headline [covid severe] * wd disgust | 1.30 | -9.23 – 11.82 | .809 |  |  |
| headline [covid severe] * DS-R | -37.18 | -125.69 – 51.32 | .410 |  |  |
| DS-R * wd disgust | 8.68 | 0.45 – 16.91 | .039 | ***** |  |
| DS-R * headline [covid severe] * wd disgust | -3.77 | -14.45 – 6.90 | .488 |  |  |
| **Random Effects** | | | | | |
| σ2 | 75495.15 | | | | |
| τ00 word | 4508.44 | | | | |
| τ00 participant | 32400.94 | | | | |
| ICC | 0.33 | | | | |
| N participant | 69 | | | | |
| N word | 99 | | | | |
| Observations | 5869 | | | | |
| Marginal R2 / Conditional R2 | 0.141 / 0.423 | | | | |

**Appendix 4.** LMMs for DS-R and word valence

**Summary of the linear mixed-effects model with reciprocally transformed RTs as a dependent variable.** Model’s formula: recipr.rt ~ log.freq + length + concreteness + AoA + ns + if.match + wd.arousal + DS-R*headline*wd. valence + (1 | participant) + (1 | word). Asterisks indicate significance (***<.001, **<.01, *<.05, .<.07).

| *Predictors* | *Estimates* | *CI* | *p* |  |
| --- | --- | --- | --- | --- |
| (Intercept) | -836.53 | -975.36 – -697.69 | <.001 | ******* |
| log freq | -42.80 | -54.48 – -31.12 | <.001 | ******* |
| length | 6.81 | -0.61 – 14.22 | .072 |  |
| concreteness | 15.08 | -1.77 – 31.93 | .079 |  |
| AoA | 55.81 | 38.79 – 72.83 | <.001 | ******* |
| ns [ns] | -193.43 | -287.64 – -99.22 | <.001 | ******* |
| if match [mismatch] | -24.17 | -110.82 – 62.47 | .584 |  |
| wd arousal | 5.15 | -26.01 – 36.31 | .746 |  |
| wd valence | 8.96 | -3.19 – 21.12 | .148 |  |
| headline [covid severe] | -46.01 | -132.48 – 40.46 | .297 |  |
| DS-R | 18.50 | -51.04 – 88.03 | .602 |  |
| headline [covid severe] * wd valence | 1.31 | -7.89 – 10.51 | .780 |  |
| headline [covid severe] * DS-R | -35.99 | -124.35 – 52.36 | .425 |  |
| DS-R * wd valence | -6.86 | -14.02 – 0.30 | .060 | **.** |
| DS-R * headline [covid severe] * wd valence | 8.30 | -1.06 – 17.67 | .082 |  |
| **Random Effects** | | | | |
| σ2 | 75530.79 | | | |
| τ00 word | 4463.52 | | | |
| τ00 participant | 32365.10 | | | |
| ICC | 0.33 | | | |
| N participant | 69 | | | |
| N word | 99 | | | |
| Observations | 5869 | | | |
| Marginal R2 / Conditional R2 | 0.140 / 0.422 | | | |

**Appendix 5.** LMMs for W-P and word disgust

**Summary of the linear mixed-effects model with reciprocally transformed RTs as a dependent variable.** Model’s formula: recipr.rt ~ log.freq + length + concreteness + AoA + ns + if.match + wd.arousal + W-P*headline*wd. disgust + (1 | participant) + (1 | word). Asterisks indicate significance (***<.001, **<.01, *<.05, .<.07).

| *Predictors* | *Estimates* | *CI* | *p* |  |
| --- | --- | --- | --- | --- |
| (Intercept) | -831.35 | -972.44 – -690.26 | <.001 | ******* |
| log freq | -41.76 | -53.27 – -30.25 | <.001 | ******* |
| length | 6.02 | -1.39 – 13.42 | .111 |  |
| concreteness | 20.04 | 2.66 – 37.43 | .024 | * |
| AoA | 59.13 | 41.01 – 77.26 | <.001 | ******* |
| ns [ns] | -207.92 | -317.98 – -97.86 | <.001 | ******* |
| if match [mismatch] | -28.49 | -117.98 – 61.00 | .533 |  |
| wd arousal | 5.20 | -26.51 – 36.91 | .748 |  |
| wd disgust | -6.00 | -20.65 – 8.65 | .422 |  |
| headline [covid severe] | -44.22 | -132.51 – 44.08 | .326 |  |
| W-P | 6.98 | -63.11 – 77.07 | .845 |  |
| headline [covid severe] * wd disgust | -4.33 | -15.04 – 6.37 | .427 |  |
| headline [covid severe] * W-P | -25.63 | -115.86 – 64.60 | .578 |  |
| W-P * wd disgust | 16.51 | 9.06 – 23.96 | <.001 | ******* |
| W-P * headline [covid severe] * wd disgust | -6.75 | -17.40 – 3.90 | .214 |  |
| **Random Effects** | | | | |
| σ2 | 75240.70 | | | |
| τ00 word | 4528.23 | | | |
| τ00 participant | 32642.13 | | | |
| ICC | 0.33 | | | |
| N participant | 69 | | | |
| N word | 99 | | | |
| Observations | 5869 | | | |
| Marginal R2 / Conditional R2 | 0.142 / 0.426 | | | |

**Appendix 6.** LMMs for W-P and word valence

**Summary of the linear mixed-effects model with reciprocally transformed RTs as a dependent variable.** Model’s formula: recipr.rt ~ log.freq + length + concreteness + AoA + ns + if.match + wd.arousal + W-P*headline*wd. valence + (1 | participant) + (1 | word). Asterisks indicate significance (***<.001, **<.01, *<.05, .<.07).

| *Predictors* | *Estimates* | *CI* | *p* |  |
| --- | --- | --- | --- | --- |
| (Intercept) | -825.73 | -967.46 – -683.99 | <.001 | ******* |
| log freq | -42.69 | -54.37 – -31.02 | <.001 | *** |
| length | 6.79 | -0.62 – 14.21 | .073 |  |
| concreteness | 15.18 | -1.67 – 32.02 | .077 |  |
| AoA | 55.92 | 38.90 – 72.93 | <.001 | *** |
| ns [ns] | -207.32 | -317.20 – -97.44 | <.001 | *** |
| if match [mismatch] | -28.56 | -117.90 – 60.78 | .531 |  |
| wd arousal | 5.00 | -26.15 – 36.16 | .753 |  |
| wd valence | 6.45 | -5.80 – 18.71 | .302 |  |
| headline [covid severe] | -42.45 | -130.49 – 45.58 | .345 |  |
| W-P | 1.01 | -68.89 – 70.92 | .977 |  |
| headline [covid severe] * wd valence | 4.18 | -5.15 – 13.50 | .380 |  |
| headline [covid severe] * W-P | -23.00 | -112.98 – 66.98 | .616 |  |
| W-P * wd valence | -10.16 | -16.59 – -3.72 | .002 | ****** |
| W-P * headline [covid severe] * wd valence | 3.89 | -5.40 – 13.17 | .412 |  |
| **Random Effects** | | | | |
| σ2 | 75409.13 | | | |
| τ00 word | 4462.91 | | | |
| τ00 participant | 32530.62 | | | |
| ICC | 0.33 | | | |
| N participant | 69 | | | |
| N word | 99 | | | |
| Observations | 5869 | | | |
| Marginal R2 / Conditional R2 | 0.141 / 0.424 | | | |
