# Supplementary material for: COVIDisgust: Language processing through the lens of partisanship
Source: PLoS One. 2022 Jul 21;17(7):e0271206. doi: 10.1371/journal.pone.0271206 (PMC9302854; doi:10.1371/journal.pone.0271206)
Supplement: Attachment — Submitted filename: Response to Reviewers Comments.docx [file pone.0271206.s003.docx]

**Response to the Editor’s:**

(1) Your motivation for the GAMM
You stated your motivation for the GAMM analysis as "Since ratings on a Likert-type scale yield ordinal data and thus, in principle, should not be analyzed with Gaussian family models assuming continuous data, we additionally ran a generalized additive mixed-modeling analysis (GAMM) for ordinal data" (p. 12). However, given this motivation, it might sound puzzling to the readers why you are not analyzing the data with something like the cumulative-link mixed-effects model for ordinal data (with the R package "ordinal"). I am not asking you to redo the whole analyses, but I would like you to reconsider your motivation for the GAMM. Doesn't your motivation have something to do with potential nonlinearity?

We have changed the text of the manuscript to “Initially the data was analyzed using generalized additive mixed modeling (GAMM) for ordinal data following (60). However, the resulting analysis was highly complex, and it was advised that we use linear mixed modeling instead. The results of the two analyses were virtually identical, and we will thus report linear mixed modeling for simplicity. The full GAMM analysis with the scripts and plots is available for review on OSF.” We hope this wording will be less confusing.

(2) RTs
The way you transformed RTs (after the reciprocal transformation) is not common, I personally think. Given that the resulting RTs are still different from the original RTs, not all readers might agree with your statement that "the plots can be read intuitively" (p. 24). However, I am not asking you to redo the analyses because it is not wrong either. I would just like you to explore optimal reporting method in the future. If an intuitive interpretation is what you want to achieve, I think a back-transformation can be applied when plotting the model-predicted values.

> RT = c(600, 700, 800) # for these sample RTs
> (-1000/RT)*1000 # this was done in this study
  # [1] -1666.667 -1428.571 -1250.000
> -1000/(-1000/RT) # back-transformation for the reciprocal transformation
  # [1] 600 700 800

Thank you! We will certainly keep it in mind for future analyses. To clarify, the comment about intuitive reading meant that, after this transformation, shorter RTs are still plotted in the bottom of the Y-axis and longer RTs at the top of the Y-axis, but we do see your point here.

(3) For all p-values and rs, please remove the zero before the decimal point.

We have removed the zeroes.

(4) This might be my problem, but it seems that the interpretation for the W-P scale is not provided before the text "More liberal participants rated the stimuli as more disgusting..." (p. 15) It is therefore not clear, for "liberal," which part of the scale the reader should focus on.

We have added the following clarification: “More liberal participants (lower W-P score)...”

(5) "The lines for the five levels of word disgust are..." (p. 16) might be misleading because it is not a factor with five levels, isn't it? Do you mean quantiles?

Thank you, we have corrected the error.

(5) Please double-check your statement on page 19: "The interaction between the participant's political ideology and valence ratings." Do you mean an interaction with word valence?

Thank you for spotting this, we have corrected this error.

**Response to Reviewer 1:**

Reviewer #1: Generally speaking, the authors revised as the reviewers suggested. However, there are some minor points that need to be explained or revised.

1.Line 14-16: In the abstract, I was wondering if the detailed reference information should be given in the parenthesis. My suggestion is to remove it.

We have removed the detailed reference.

2.Line 25: “after the headlines emphasizing it” seems ungrammatical. Please confirm.

We have changed the sentences to “More liberal participants assigned higher disgust ratings after the headlines *discounted* the threat of COVID-19, whereas more conservative participants did so after the headlines *emphasized* it.” for more clarity.

3.In the section of “Introduction” and “Discussion”, I suggest adding some subtitles so that the ideas are more clearly elaborated. Furthermore, the ideas expressed in these two sections should be more symmetrical, being more focused on the hypotheses of the research.

We have added subtitles and restructured the Introduction and the General Discussion for more clarity.

**Response to Reviewer 3:**

Reviewer #3: Comments to the authors

The manuscript was well revised including additional explanations for materials and procedures as reviewers requested. The added explanations of the data analysis on page 11 sound convincing, and I respect how the authors changed the statistical analysis during the revision.
However, the manuscript is still challenging for readers to understand, and it probably requires readers to read several times to fully comprehend what the authors are trying to do.
Comments with (>>) below are some minor revisions and I hope that they could enhance the readers' comprehensibility of the manuscript.

p.7 line 134
untrustworthy, and be discarded by more conservative participants, resulting in no effect.

>>The authors could specify to which valuable (disgust rating?) the effect is going to be less influential.

We clarified the sentence: “Headlines emphasizing the threat will be considered uncredible, untrustworthy, and be discarded by more conservative participants, resulting in no effect in disgust ratings.”

p.28 line 515
Importantly, we did not find an effect of the headline on word recognition latencies. This suggests that fluctuating levels of disgust may not affect such core language processing mechanisms as lexical access. It may also mean that stable traits are more predictive of lexical recognition latencies than fluctuating states.

>>This explanation of the results of experiment 2 (LDT) is convincing. However, the result of no effect of COVID headlines makes readers confused a little bit.
Political ideology has an effect on online language (disgust words) processing, not limited to pandemic associated topics, is that what the authors are trying to say?
Probably additional sentences are needed to help readers understand the interpretation of the results and how the results can be generalized.

We have rewritten the paragraph and added more details to both the Discussion after Exp 2 and the General Discussion. Specifically, we have added a subsection “Differential effects of traits and states on lexical access” to General Discussion.
